# Marital status and living apart affect sleep quality in male military personnel: a study of the China’s Navy during COVID-19

**DOI:** 10.3389/fpsyt.2023.1178235

**Published:** 2023-07-27

**Authors:** Xin Guo, Yao Meng, Hao Lian, Yinan Li, Ying Xu, Ruike Zhang, Jingzhou Xu, Hao Wang, Shuyu Xu, Wenpeng Cai, Lei Xiao, Tong Su, Yunxiang Tang

**Affiliations:** ^1^Department of Medical Psychology, Faculty of Psychology, Naval Medical University, Shanghai, China; ^2^Department of Diving and Hyperbaric Medical Research, Naval Medical Center, Naval Medical University, Shanghai, China; ^3^Faculty of Psychology, Naval Medical University, Shanghai, China

**Keywords:** daytime sleepiness, dysfunctional sleep beliefs, marital status, military personnel, sleep quality

## Abstract

**Background:**

Marital status is a robust sociodemographic predictor of sleep. Having to live apart from spouse may have different implications than those of cohabitants or singles, especially in military personnel. Further research on this group will help provide knowledge in advance and facilitate early targeted interventions.

**Methods:**

An online questionnaire study was conducted from July to November 2021. A total of 1,832 male military personnel completed the questionnaire. The marital status was measured by a self-reported single choice question. Pittsburgh sleep quality index (PSQI), Epworth Sleepiness Scale (ESS) and The Dysfunctional Beliefs and Attitudes about sleep scale (DBAS-16) were used to measure sleep-related outcomes. Inverse probability weighting (IPW) was applied to reduce the effects of confounding. Logistic regression was used to analyze the relationship between marital status and sleep and explore the impact of living together or not.

**Results:**

After inverse probability weighting, the prevalence of poor sleep quality, sleepiness and dysfunctional beliefs were 16.1, 20.1 and 7.1%, respectively. One-way ANOVA results for the means of both groups were statistically significantly different, except for the sleep latency and sleep disturbance dimensions of PSQI. Participants who were married were more likely to have poor sleep quality (OR: 1.408, 95% CI: [1.10, 1.80]), to have daytime sleepiness (OR: 1.560, 95% CI: [1.27, 1.92]) and to develop dysfunctional beliefs and attitudes (OR: 2.497, 95% CI: [1.65, 3.80]) than those who were unmarried. Further analysis showed that the odds of developing poor sleep quality and DBAS in participants who married but living apart were significantly bigger than those unmarried (OR: 1.548 and 3.991, respectively.), while there were no significant differences in the odds of daytime sleepiness (OR: 0.738, *p* = 0.050). Age was a protective factor for the development of bad sleep outcomes, while family economic was an independent risk factor.

**Conclusion:**

Marital status appear important for sleep quality, daytime sleepiness and sleep beliefs. The effect of living apart or not should be considered separately as an important predictor of sleep.

## Introduction

Sleep loss is a common problem for military personnel, and cognitive (1, 2) (such as memory, attention, alertness, executive function, etc.) and physical deficits (3) (such as aerobic capacity, muscle strength, and military specific performance, etc.) that result from sleep loss may lead to serious consequences (3). Research has shown that the prevalence of insomnia in the U.S. Army before deployment is 19.9%. Enlisted personnel were five times more likely to report insomnia than officers (4). Moreover, military personnel are often required to operate continuously in training or deployed environments (5), and in many cases the mission is urgent and requires a high level of concentration. Dangerous combat environments, stressors and limited sleep time make it difficult for military personnel to adequately recover from missing sleep (2, 6). A recent meta-analysis found that sleep quality in Navy showed a progressive trend of getting worse before COVID-19 (7). What’s more, military personnel showed a higher prevalence of insomnia during the pandemic compared to the pre-pandemic period (8). In the post-epidemic era, where the impact of quarantine is diminishing while the uncertainty of the epidemic is still intensifying, the sleep status of military personnel may face new challenges (9, 10). In addition, a research study on veterans showed that changes in social support and family relationships were critical to the onset of insomnia symptoms during the epidemic (11). This prompts us to pay special attention to sociodemographic factors that affect sleep. Prevention and promotion programs should be developed to reverse this negative trend in the military, especially during COVID-19 (12).

The underlying sociodemographic risk factors for developing sleep disorders are critical because officials can know beforehand which populations are at a higher risk for certain sleep disorders and implement early interventions for those at risk (13). The effects of sociodemographic factors, such as age, sex, alcohol intake, and race on sleep, have been studied in the military population (4, 14–16). However, the effect of marital status, a robust sociodemographic predictor of health and well-being (17, 18), has not been adequately studied among military personnel.

Many studies have shown that marital status is an important factor in sleep and maybe a major mechanism for health problems (19–21), but little is known about this relationship for military personnel, for whom marriage does not mean co-habitation, and it is common to face separation due to the demands of mission or deployment. Moreover, studies on the effects of marital status on sleep quality are ambiguous (22). On the one hand, being married appears to improve sleep quality. For example, a research on a sample of Hispanics/Latinos found that being married or cohabiting was associated with better sleep health overall, including having a normal sleep schedule, fewer insomnia symptoms and higher sleep efficiency (23). This overall better sleep performance may be related to increased REM sleep when co-sleeping with a partner (24). On the other hand, being married or cohabiting may also bring sleep problems. It is reported that spouses of snorers report more frequent sleep problems, insomnia, daytime fatigue and drowsiness (25). Studies focused on married women have found that married mothers have less time for sleep compared to other mothers (19), while another study reported that co-sleeping with a partner was associated with longer sleep and more awakenings (26). In addition, based on previous studies, we have also found that the impairment of romantic relationships, such as widowhood, separation, or divorce, has been linked to greater instances of sleep disturbances (22). A follow-up study of middle-aged and older rural populations found that those who were separated/divorced/widowed/never married exhibited poorer sleep, which in turn increased the odds of depression (27).

There is a relative paucity of research on marital status and sleep in the military, with most of the existing studies addressing marital quality between veterans and their spouses (28). In fact, the study of marital status has important applications because it is much easier to obtain the marital status of officers and soldiers in the military population than to obtain their marital quality. Studying the effect of marital status on sleep can help unit commanders target the condition of officers and soldiers for timely intervention. Moreover, military personnel who are married but living separately are common in the Chinese military community, and separation from spouses may cause plenty of problems, making their marriage at more risk than others (29). A survey of United States military couples also showed that separation of male service members from their wives increased the odds of marital instability for couples (30). Having to live apart from one’s spouse due to deployment may have different experiences than those of cohabiting or single individuals, so studying these service members separately from other groups may yield different results.

Given the specificity of marital status among military personnel and the crucial role of marriage in sleep, this study intends to investigate the role of marital status on sleep quality, daytime sleepiness, and sleep beliefs among male military personnel, which will fill the gap in this field and provide a theoretical basis for targeted sleep interventions for troops to improve sleep quality and operational capability in the future. To investigate the relationship between marital status and sleep, we chose the sleep scale based on its widespread use in research and clinical practice and its relevance in investigating the effects of marital status (2). Dysfunctional beliefs and attitudes about sleep (DBAS) mainly refer to misperceptions and desperate fears about the negative consequences of insomnia and other sleep problems (31, 32), which are often used as targets of cognitive behavioral therapy to improve sleep because of their operability. These beliefs may include: they need a less realistic number of hours of sleep, believe that insomnia can have a significant negative impact on their lives, and maintain the tendency to blame poor sleep on external stable causes (33). The effect of different marital statuses on dysfunctional beliefs can result in emotional problems or behavioral disorders (34), but there is a paucity of relevant research on sleep beliefs among people with different marital status. It is worth noting that controlling for sociodemographic and health factors known to influence sleep quality is critical to understanding whether marital status, a single variable, has effects beyond these known predictors. Accordingly, we used inverse probability weighting (IPW) techniques, which are increasingly used in observational studies to control for confounders, as traditional multivariate regression methods may be biased by overfitting when the number of events is small (35).

We hypothesized that married in comparison to unmarried individuals would have better self-reported sleep quality, as previous studies tend to show that marriage makes people healthier due to adequate sleep duration and fewer poor sleep beliefs (36). However, we did not make explicit hypotheses about the effect of living together on sleep outcomes because this part of the analysis is essentially exploratory in nature. Previous studies have not provided sufficient clues about the effect of living together on sleep outcomes in military personnel.

## Methods

### Participants

The participants in this study were recruited from two naval units in China. An online questionnaire study was conducted from July to November 2021 using convenience sampling. Based on the sample size and resource limitations of previous similar studies, 1,832 military personnel completed the questionnaire. In addition, we used the method of inverse probability weighting, which allowed for improved test validity of the sample. However, 48 patients were excluded from subsequent analyses. Specific reasons for exclusion included (a) unclear age (*N* = 2), (b) age younger than 18 years (*N* = 4), and (c) current concomitant illnesses that may affect sleep (*N* = 42).

The final sample size was 1,784 and the questionnaire validity rate was 97.38%. All the participants were male, considering that military personnel are predominantly male, such a data composition is acceptable. This study was approved by the Medical Research Ethics Committee of Naval Medical University (protocol number 20210310041) and approval number NMUMREC-2021-041, and all participants provided informed consent through an online system.

### Measures

#### Marital status

In the Chinese military community, unmarried personnel live in group quarters, while married personnel have the opportunity to live out even separately from their spouses. Considering the specificity of the marital status of the military population, participants’ options regarding marital status included unmarried, married (married living together or married living apart), divorced, and widow (er). Notably, no participants chose “divorced” or “widowed,” so these two items were not included in the analysis of this paper.

#### Confounding variables

A self-designed demographic characteristics questionnaire was used to investigate information that could be confounding variables, including age, gender, education level, marital status, body mass index (BMI), family residence, family relationship, family economy, whether he is the only child in his family or not, whether the family of origin is complete or not, self-assessment stress from family, comrade relationship, shift work, and average overtime days per week. Among them, BMI = weight (kg)/(height (m)*height), family relationship, and family economy were determined using a five-point Likert scale score from “very poor” to “very good.” The answer to the stress from family was determined by the answer from 1 (no pressure) to 4 (severe pressure). Besides, the comrade relationship was determined by a single question, with answers ranging from 1 (no stress/very bad relationship) to 10 (extreme stress/very good). Other data were obtained using the corresponding single selections.

#### Pittsburgh sleep quality index

The Pittsburgh sleep quality index (PSQI) is a 19-item self-rating scale that is commonly utilized to reflect the sleep quality of participants over the past few months (37). The reliability of the Chinese version of the PSQI has been confirmed in the military population (38). In this study, it was utilized to assess the sleep quality of the participants. The 19 items of the PSQI are divided into seven dimensions: subjective sleep quality (item 6), sleep latency (items 2 and 5a), sleep duration (item 4), sleep efficiency (items 1, 3, and 4), sleep disturbance (items 5b–5j), use of sleep medication (item 7), and daytime dysfunction (items 8 and 9). The score of each dimension was 0–3, and the scores of the seven dimensions were summed to obtain the total PSQI score, ranging from 0 to 21, with higher scores indicating poorer sleep quality. A total PSQI score ≥ 7 indicated poor sleep quality and < 7 indicated good sleep quality.

#### The Epworth sleepiness scale

Epworth sleepiness scale (ESS) is a self-rating scale designed to assess daytime sleepiness. Originally developed by Johns (39), it can be used as a screening tool to identify individuals with excessive daytime sleepiness and potential sleep disorders. Participants self-assessed their likelihood of dozing off in eight different daily situations on a four-point Likert scale. Responses were scored from 0 (“Never doze off”) to 3 (“Likelihood of dozing off is high”). Participants were asked to rate themselves based on “how you usually doze off.” The total ESS score for a total of eight questions ranged from 0 to 24, with ≤10 being normal, 11–15 being suspicious sleepiness, and ≥ 16 being severe sleepiness. The scale has high internal consistency and ranges from 0.73 to 0.88, as measured by Cronbach’s alpha (39).

#### The dysfunctional beliefs and attitudes about sleep scale

The dysfunctional beliefs and attitudes about sleep scale (DBAS-16), originally designed by Morin et al., was developed to determine the types of personal beliefs and attitudes about sleep (40). The scale has been gradually used in academic research and clinical diagnoses worldwide and has been translated into several languages, including Chinese (41, 42). The reliability and validity of the Chinese version of the DBAS-16 have been validated (43). In this study, the DBAS-16 was utilized to assess beliefs and attitudes about sleep. The scale comprises four dimensions: (1) consequences of insomnia (items 5, 7, 9, 12, and 16), (2) worry/helplessness about sleep (items 3, 4, 8, 10, 11, and 14), (3) expectations about sleep (items 1 and 2), and (4) medication (items 6, 13, and 15). Each item is rated on a scale ranging from 1 (“strongly agree”) to 5 (“strongly disagree”). The total score on the DBAS-16 is the sum of the scores for all 16 items, ranging from 16 to 80. The higher the level of dysfunctional beliefs of a participant, the lower the total score. An average DBAS-16 score < 2.0, which is considered the level of unhelpful beliefs associated with clinically significant insomnia (44, 45).

### Statistical analysis

Categorical variables are presented as frequencies and percentages, and continuous variables are presented as mean ± standard deviation (SD). Differences in continuous and categorical variables across marital groups were tested using analysis of variance (ANOVA) and Chi-squared test, respectively. The differences between married and unmarried individuals were statistically analyzed. To estimate the causal effect of marital status on sleep quality among military personnel, we employed a weighting approach. Inverse probability weighting (IPW) was used to reduce the effects of confounding variables and maximize exchangeability among different marital groups (46, 47). Exchangeability means that different groups have similar characteristics; therefore, they also have a similar risk of being married and unmarried. Only when different groups are exchangeable can the outcome differences between the two groups be attributed to the effect of exposure. To reduce the effect of selection bias and potential confounding factors, differences in baseline characteristics (age, education level, BMI, family residence, family relationship, family economy, whether an only child or not, growing family, stress from family, comrade relationship, shift work, or not) were adjusted using weighted generalized linear mixed models.

Logistic regression was used to examine the effect of marital status on sleep-related outcomes. The reference groups for the other items were annotated in the results. After obtaining preliminary results for married and unmarried personnel, we conducted a further analysis to explore the differences between unmarried and married personnel living together, as well as unmarried and married personnel living separately. We extracted the two groups of married data and compared them separately with the unmarried group. The logistic regression procedure was the same as the analysis process mentioned above.

Statistical Package for the Social Sciences software (version 26.0; SPSS, Chicago, IL, United States) and R software (version 4.2.0, Package “ipw” and “cmprskcoxmsm”) were employed for all statistical analyses, and statistical significance was set at *p* < 0.05.

## Results

### Demographic characteristics

Table 1 shows the descriptive characteristics of the study participants by the marital group before and after IPW. Among the 1,784 participants (mean age = 26.14 ± 5.821 years), the majority were unmarried (*n* = 1,431 [80.2%]), 353 (19.8%) were married, 147 (8.2%) were married but living apart, and 206 (11.5%) were married and living together. Before the IPW process, the two marital status groups differed significantly in their demographic data. Statistically significant differences were found across age, education level, BMI, family residence, family relationship, family economy, stress from family, comrade relationship, and shift work or not.

**Table 1:** Descriptive characteristics of the sample by marial status before and after IPW.

Group	Level	Unmatched			IPW		
		Unmarried	Married	*p*	Unmarried	Married	*p*
*N*		1,431	353		2164.99	991.57	
age [mean (SD)]		22.04 (2.62)	31.57 (4.66)	<0.001	25.75 (5.87)	27.36 (4.53)	0.270
Education level (%)	High school or below	432 (30.2)	63 (17.8)	<0.001	467.0 (21.6)	300.1 (30.3)	0.497
	Junior college	719 (50.2)	150 (42.5)		1004.6 (46.4)	334.9 (33.8)	
	Bachelor or above	280 (19.6)	140 (39.7)		693.4 (32.0)	356.5 (36.0)	
BMI [mean (SD)]		22.49 (2.11)	24.17 (2.21)	<0.001	22.79 (1.91)	23.31 (1.96)	0.064
Family residence (%)	Rural areas	872 (60.9)	184 (52.1)	0.003	1215.8 (56.2)	626.7 (63.2)	0.554
	Urban areas	559 (39.1)	169 (47.9)		949.2 (43.8)	364.9 (36.8)	
Family relationship (%)	Not very good	157 (11.0)	78 (22.1)	<0.001	328.5 (15.2)	175.1 (17.7)	0.743
	Very good	1,274 (89.0)	275 (77.9)		1836.5 (84.8)	816.5 (82.3)	
Family economic (%)	Very poor	67 (4.7)	26 (7.4)	0.002	159.1 (7.3)	62.4 (6.3)	0.430
	Poor	324 (22.6)	90 (25.5)		464.2 (21.4)	216.0 (21.8)	
	General	874 (61.1)	217 (61.5)		1360.6 (62.8)	564.4 (56.9)	
	Good	101 (7.1)	17 (4.8)		107.8 (5.0)	35.9 (3.6)	
	Very good	65 (4.5)	3 (0.8)		73.2 (3.4)	112.8 (11.4)	
The only child or not (%)	No	821 (57.4)	207 (58.6)	0.71	1453.8 (67.1)	559.4 (56.4)	0.308
	Yes	610 (42.6)	146 (41.4)		711.2 (32.9)	432.2 (43.6)	
Growing family (%)	Family integrity	1,277 (89.2)	320 (90.7)	0.497	1980.1 (91.5)	920.3 (92.8)	0.638
	Single parent or divorced	154 (10.8)	33 (9.3)		184.9 (8.5)	71.3 (7.2)	
Stress from family [mean (SD)]	No pressure	774 (54.1)	76 (21.5)	<0.001	961.4 (44.4)	464.3 (46.8)	0.951
	Mild stress	425 (29.7)	168 (47.6)		730.2 (33.7)	302.6 (30.5)	
	Moderate pressure	205 (14.3)	97 (27.5)		434.0 (20.0)	207.8 (21.0)	
	Severe pressure	27 (1.9)	12 (3.4)		39.4 (1.8)	16.8 (1.7)	
Comrade relationship [mean (SD)]		1.52 (2.38)	1.98 (2.20)	0.001	1.99 (2.60)	2.39 (2.78)	0.509
Shift work or not (%)	No	1,221 (85.3)	223 (63.2)	<0.001	1665.0 (76.9)	633.7 (63.9)	0.234
	Yes	210 (14.7)	130 (36.8)		500.0 (23.1)	357.9 (36.1)	

After IPW, these demographic variables became balanced and comparable at *p* > 0.05, indicating that there were no significant differences in the demographic factors between the married and unmarried groups.

### Prevalence of poor sleep quality, sleepiness, and DBAS

The prevalence of poor sleep quality, sleepiness, and dysfunctional beliefs and attitudes about sleep after IPW is shown in Table 2. Prevalence results prior to IPW can be found in Supplementary Table S1. After inverse probability weighting, the mean PSQI was 4.05 (SD = 2.99). The total score of 508 in 3,157 participants was over the cut-off score of 7 points, indicating that the prevalence of poor sleep quality was 16.1%. The average scores and standard deviations for the seven dimensions are presented in Table 2. The prevalence of sleepiness was 20.1%, including 16.3% suspicious sleepiness and 3.8% severe sleepiness, with a mean score of 6.28 (SD = 4.95). The scores of 7.1% of participants showed that they had dysfunctional beliefs associated with clinically significant insomnia. The mean scores for the total DBAS-16, consequences of insomnia, worry/helplessness about sleep, expectations for sleep, and medication were 50.80, 15.65, 19.85, 3.95, and 11.35, respectively. We also calculated the mean score for each topic of DBAS-16, which was 3.18 (SD = 0.78), to facilitate comparison with results from other literature.

**Table 2:** Prevalence and mean scores of sleep-related outcomes by marital status.

	Total	Unmarried	Married	*p*
*N*	3156.6	2165.0	991.6	
Prevalence of poor sleep quality (%)	507.7 (16.1)	315.4 (14.6)	192.3 (19.4)	0.001
Total PSQI	4.05 (2.99)	4.14 (2.95)	3.85 (3.05)	0.010
Subjective sleep quality	0.75 (0.62)	0.73 (0.63)	0.81 (0.60)	<0.001
Sleep latency	0.84 (0.74)	0.84 (0.73)	0.85 (0.77)	0.727
Sleep duration	0.61 (0.59)	0.65 (0.55)	0.52 (0.67)	<0.001
Sleep efficiency	0.41 (0.77)	0.44 (0.78)	0.34 (0.74)	0.001
Sleep disturbance	0.60 (0.57)	0.61 (0.57)	0.57 (0.56)	0.137
Use of sleep medication	0.06 (0.36)	0.08 (0.40)	0.02 (0.21)	<0.001
Daytime dysfunction	0.78 (0.85)	0.80 (0.88)	0.74 (0.77)	0.038
Prevalence of sleepiness (%)	635.7 (20.1)	410.7 (19.0)	225.0 (22.7)	0.016
Prevalence of severe sleepiness (%)	118.7 (3.8)	104.6 (4.8)	14.1 (1.4)	<0.001
Total ESS	6.28 (4.95)	6.15 (5.19)	6.58 (4.38)	0.023
Prevalence of DBAS (%)	224.5 (7.1)	83.1 (3.8)	141.4 (14.3)	<0.001
Average score of DBAS-16	3.18 (0.78)	3.24 (0.69)	3.03 (0.94)	<0.001
Total DBAS-16	50.8 (12.49)	51.88 (11.03)	48.46 (14.96)	<0.001
Consequences of insomnia	15.65 (4.49)	15.92 (4.15)	15.07 (5.13)	<0.001
Worry/helplessness about sleep	19.85 (5.31)	20.23 (4.88)	19.04 (6.06)	<0.001
Expectations for sleep	3.95 (1.65)	4.15 (1.57)	3.51 (1.73)	<0.001
Medication	11.35 (2.75)	11.58 (2.27)	10.85 (3.53)	<0.001

Significant differences were evident between the groups in terms of the prevalence of poor sleep quality, daytime sleepiness, and dysfunctional beliefs and attitudes about sleep. Furthermore, one-way ANOVA results for the means of both groups were significantly different, except for the sleep latency and sleep disturbance dimensions of the PSQI.

### Marital status and sleep-related outcomes

**Figure 1:**
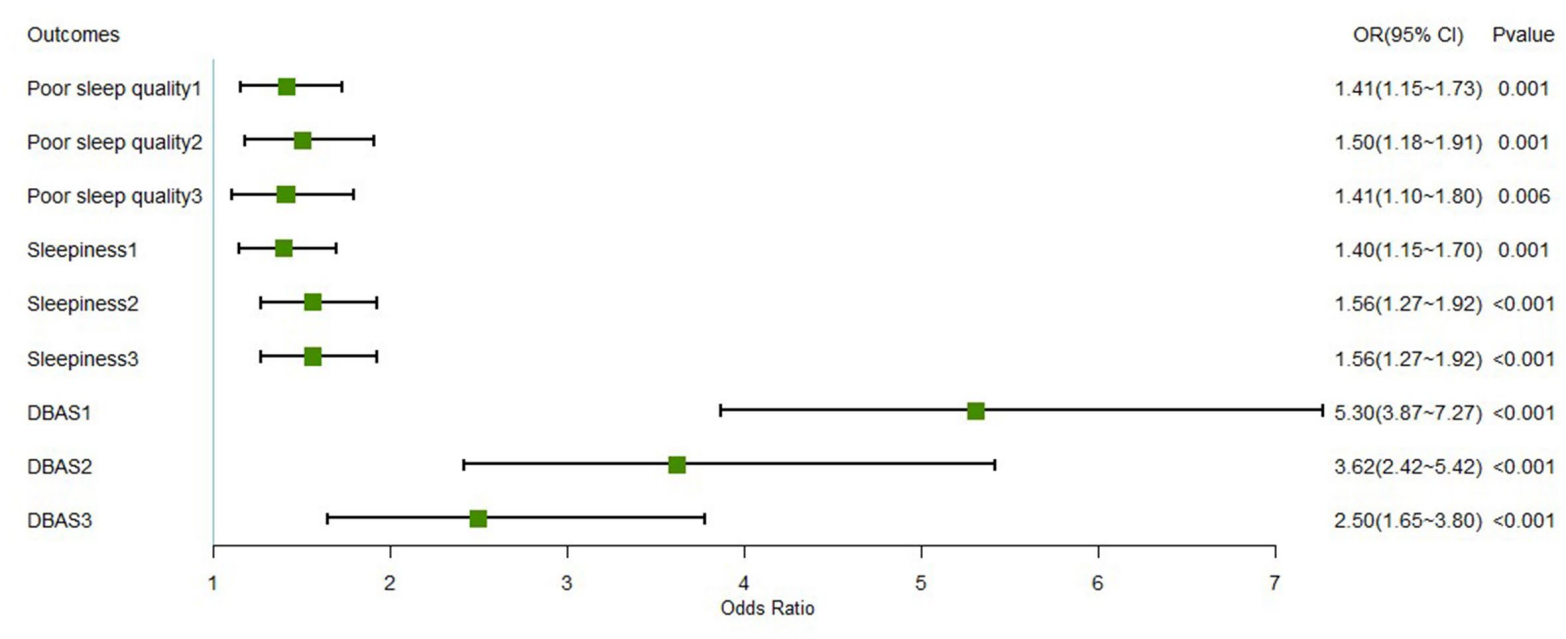
Forest plot of marital status for each sleep-related outcome. *DBAS, dysfunctional beliefs and attitudes about sleep. The covariates of each outcome indicator were added sequentially in three times, (1) represents the inclusion of demographic factors; (2) represents the inclusion of demographic factors and family-related factors; and (3) represents the inclusion of demographic variables, family-related variables, and work-related variable.

Next, we examined the relationship between marital status and three sleep-related outcomes. The outcomes of the logistic regression are given in Table 3, wherein the outcome variables included PSQI, ESS, and DBAS-16. The covariates of each outcome indicator were added sequentially three times: demographic variables, family-related variables, and work-related variables, to explore the stability of the effect of marital status on sleep. Forest plots of ORs from the three regression analyses are shown in Figure 1.

**Table 3:** Logistic regression results of association between marital status.

	Model 1: PSQI		Model 2: ESS		Model 3: DBAS-16	
	*p*	Odds ratio	*p*	Odds ratio	*p*	Odds ratio
Unmarried versus married	0.006	1.408	<0.001	1.560	<0.001	2.497
*Demographic background*						
Age	0.002	0.959	<0.001	0.909	<0.001	0.846
Educational level (ref: high school or below)	0.016		0.001		0.027	
Junior college	0.004	1.602	0.003	1.439	0.220	0.734
Bachelor or above	0.073	1.403	<0.001	1.682	0.188	1.473
BMI	0.187	0.960	<0.001	1.115	0.676	0.979
*Family factors*						
Rural areas versus urban areas	0.054	0.766	0.441	1.084	<0.001	2.312
Family relationship (other versus very good)	<0.001	0.503	<0.001	0.550	0.029	0.563
Family economic (ref: very poor)	<0.001		<0.001		<0.001	
Poor	<0.001	0.440	0.007	1.951	0.031	2.539
General	<0.001	0.331	0.014	1.808	0.282	0.613
Good	0.220	0.642	0.004	2.456	0.159	2.220
Very good	0.012	0.239	0.099	0.530	<0.001	29.179
The only child or not	0.028	1.326	0.056	1.207	0.006	0.570
Family integrity versus single parent or divorced	0.206	1.282	0.060	1.341	0.025	0.373
Stress from family (ref: no pressure)	<0.001		<0.001		0.001	
Mild stress	<0.001	7.365	<0.001	1.492	0.035	0.520
Moderate pressure	<0.001	16.735	0.469	1.109	0.045	1.721
Severe pressure	<0.001	39.809	<0.001	5.112	0.254	0.300
*Work factors*						
Comrade relationship	<0.001	1.179	0.716	0.993	<0.001	1.179
Shift work or not	<0.001	2.407	0.833	1.026	<0.001	2.601
Constant	0.166	0.342	<0.001	0.104	0.670	1.803

Model 1 showed that married participants were more likely to have poor sleep quality than those who were unmarried (OR: 1.408, 95% CI: [1.10, 1.80]), and the odds ratio remained constant during the addition of other variables. Increasing age decreases the odds of poor sleep quality, while a higher education level (junior college) is 60% more likely to generate poor sleep quality than lower education levels (high school or below). Notably, for the family relationship item, since the vast majority of participants chose “very good,” we combined the other four options and compared them together with “very good.” Participants with better family relationships and economic status were more likely to have better sleep quality than poor ones. Each increase in self-rated family stress levels substantially increases the probability of producing poor sleep quality. In addition, an increase in comradeship increases the probability of developing sleep disorders by approximately 1.4 times, while shift work increases the probability by about 18%.

The outcome variable of Model 2 is the ESS. The results showed that married military personnel were 56% more likely to have daytime sleepiness than unmarried military personnel (OR: 1.560, 95% CI: [1.27, 1.92]), and the odds ratio was also stable. Age is still a protective factor for daytime sleepiness, with each year increase in age decreasing the odds of developing daytime sleepiness by approximately 10%, whereas the higher the level of education, the greater the likelihood of daytime sleepiness. In addition, the results showed that BMI, good family finances, and greater family stress were risk factors for daytime sleepiness, while good family relationships were protective factors.

Dysfunctional beliefs and attitudes were the outcome variables in Model 3. The results showed that married male military personnel were twice as likely to develop dysfunctional beliefs and attitudes as those who were unmarried (OR: 2.497, 95% CI: [1.65, 3.80]), and the OR was always significant and at a high level, although it gradually decreased with the inclusion of other variables (see Figure 1). The association between education level and DBAS was not statistically significant, while age, good family relationships, only children, and mild family stress were protective factors. The odds of participants with better family economic levels tended to increase (OR of poor vs. very poor: 2.539; OR of very good vs. very poor: 29.179). In addition, individuals who grew up in single-parent or divorced families had fewer dysfunctional beliefs and attitudes than those with intact families (OR: 0.373). Comradeship and shift work were risk factors for developing dysfunctional beliefs and attitudes, with OR of 1.179 and 2.601, respectively.

### Living together or not and sleep-related outcomes

To further explore the effect of living apart and living together on sleep, a further analysis was conducted. We extracted two groups of married data and compared them separately from the unmarried group. Notably, in the military community, unmarried personnel live in groups within the camp.

The results of the logistic regression between the unmarried and married but living apart groups are shown in Table 4. We found that the odds of developing poor sleep quality and DBAS among participants who were married but living apart were significantly higher than in those who were unmarried (odds ratio [OR]: 1.548 and 3.991, respectively). No significant differences in the OR of daytime sleepiness were observed (OR: 0.738; *p* = 0.050). The trends of the remaining individual variables on the PSQI were the same as the results of analyses between unmarried and married individuals. In Model 5, the significance of marital status, education, and family relationships disappeared, while the variables of family integrity, family stress, and shift work changed from insignificant to significant compared to the previous stratification. Compared with unmarried people, married people living apart are approximately three times more likely to have dysfunctional sleep beliefs. In the extracted data, those with a Bachelor’s degree or higher were more likely to have dysfunctional sleep beliefs than those with a high school education or lower. The OR values for the other variables did not vary significantly.

**Table 4:** Logistic regression results of association between unmarried and married living apart.

	Model 4: PSQI		Model 5: ESS		Model 6: DBAS-16	
	*p*	Odds ratio	*p*	Odds ratio	*p*	Odds ratio
Unmarried versus married living apart	0.010	1.548	0.050	0.738	<0.001	3.991
*Demographic background*						
Age	0.006	0.956	<0.001	0.892	<0.001	0.836
Educational level (ref: high school or below)	0.067		0.586		0.005	
Junior college	0.024	1.487	0.456	1.103	0.771	0.928
Bachelor or above	0.361	1.228	0.858	0.969	0.014	2.137
BMI	0.508	0.977	<0.001	1.133	0.664	0.977
*Family factors*						
Rural areas versus urban areas	0.090	0.766	0.251	1.143	0.001	2.141
Family relationship (other versus very good)	0.001	0.552	0.108	0.774	0.004	0.457
Family economic (ref: very poor)	<0.001		0.004		<0.001	
Poor	<0.001	0.322	0.011	2.037	0.334	1.565
General	<0.001	0.258	0.021	1.898	0.171	0.511
Good	0.025	0.391	0.005	2.652	0.496	1.503
Very good	0.006	0.191	0.766	0.883	<0.001	16.766
The only child or not	0.003	1.546	0.328	1.115	0.035	0.640
Family integrity versus single parent or divorced	0.459	1.178	0.006	1.584	0.039	0.401
Stress from family (ref: no pressure)	<0.001		<0.001		0.015	
Mild stress	<0.001	6.648	<0.001	1.784	0.152	0.639
Moderate pressure	<0.001	19.387	0.029	1.427	0.069	1.696
Severe pressure	<0.001	41.677	<0.001	5.879	0.278	0.310
*Work factors*						
Comrade relationship	<0.001	1.210	0.061	1.041	<0.001	1.173
Shift work or not	<0.001	2.275	0.036	1.334	0.001	2.303
Constant	0.144	0.277	<0.001	0.080	0.387	3.449

**Figure 2:**
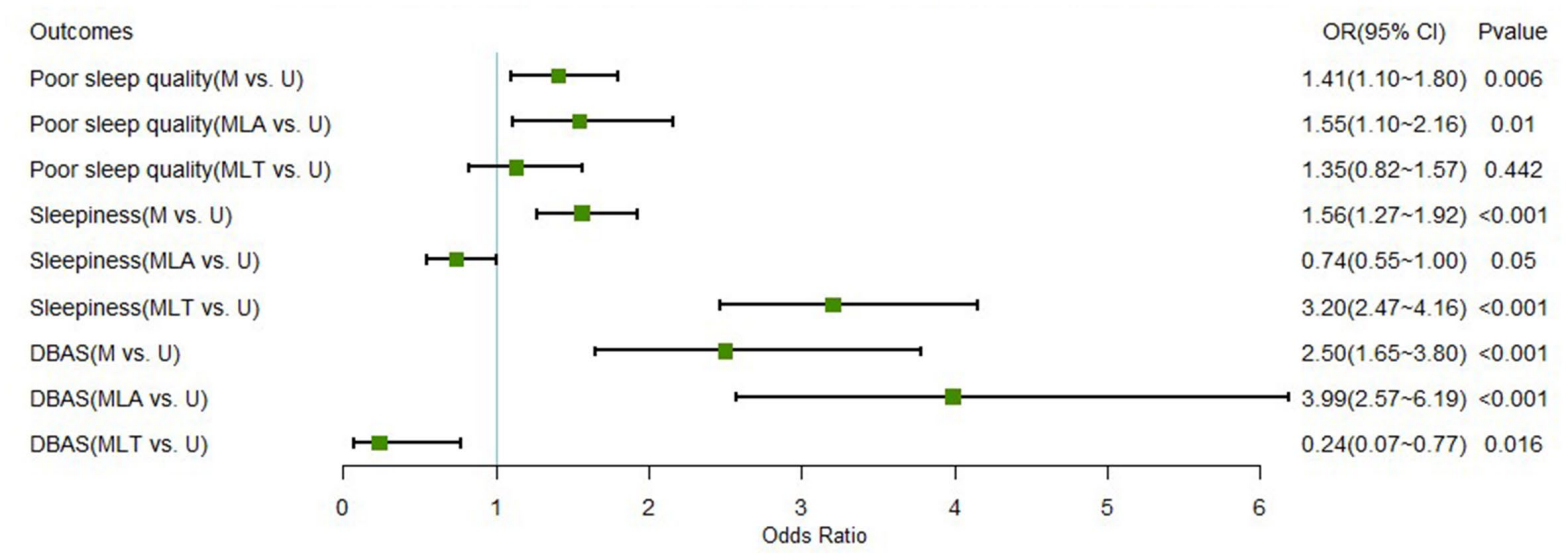
Forest plot of different sleep outcome variables between marital groups. M versus U, married versus unmarried; MLA versus U, married living apart versus unmarried; MLT versus U, married living together versus unmarried; DBAS, dysfunctional beliefs and attitudes about sleep.

Table 5 provides the findings of the logistic regression analysis between unmarried, married, and living together. In terms of sleep quality, there was no significant difference between unmarried and married living together, whereas married living together was 3.202 times more likely to produce daytime sleepiness than unmarried and 0.235 times more likely to produce dysfunctional beliefs. Figure 2 illustrates the odds ratio of developing poor sleep quality, daytime sleepiness, and dysfunctional beliefs in different groups.

**Table 5:** Logistic regression results of association between unmarried and married living together.

	Model 7: PSQI		Model 8: ESS		Model 9: DBAS-16	
	*p*	Odds ratio	*p*	Odds ratio	*p*	Odds ratio
Unmarried versus married living together	0.442	1.135	<0.001	3.202	0.016	0.235
*Demographic background*						
Age	0.010	0.964	<0.001	0.899	0.003	0.909
Educational level (ref: high school or below)	0.006		0.197		0.359	
Junior college	0.003	1.776	0.675	1.058	0.489	1.217
Bachelor or above	0.003	1.903	0.099	1.290	0.519	0.778
BMI	0.362	0.969	0.029	1.058	0.840	0.989
*Family factors*						
Rural areas versus urban areas	0.032	0.729	0.713	0.960	0.244	1.325
Family relationship (other versus very good)	<0.001	0.546	0.003	0.655	0.992	0.996
Family economic (ref: very poor)	<0.001		0.001		0.005	
Poor	<0.001	0.281	<0.001	2.702	0.587	1.273
General	<0.001	0.266	0.004	2.079	0.121	0.496
Good	0.117	0.560	<0.001	3.240	0.628	1.316
Very good	0.049	0.316	0.103	1.905	0.832	1.144
The only child or not	<0.001	1.782	0.005	1.349	0.002	2.070
Family integrity versus single parent or divorced	0.079	1.440	0.195	1.241	0.251	0.606
Stress from family (ref: no pressure)	<0.001		<0.001		0.062	
Mild stress	<0.001	5.129	0.199	1.167	0.306	0.730
Moderate pressure	<0.001	9.926	0.917	0.984	0.062	1.759
Severe pressure	<0.001	35.215	<0.001	5.385	0.684	0.656
*Work factors*						
Comrade relationship	<0.001	1.117	0.121	1.033	<0.001	1.158
Shift work or not	<0.001	2.753	0.217	1.179	0.834	0.936
Constant	0.224	0.351	0.161	0.395	0.438	0.311

## Discussion

This study explored the effect of marital status on sleep quality, daytime sleepiness, and dysfunctional beliefs and attitudes regarding sleep among the male military population. Our study was conducted during a period of well-controlled pandemics when the troops were not away on duty. Therefore, it is representative of the daily sleep quality of male Chinese military personnel. The distribution of marital status varies across demographic characteristics, and it is not difficult to understand the significant effect of age on marital status in our study group, which was overwhelmingly composed of adult males under the age of 35 years. Research has shown that military personnel marry and start families earlier than civilians (48). However, because of the demands of their jobs, they move much more often than civilians, and are often absent from their families for months at a time (48). Factors related to family (either family of origin factors or newly formed families) or work may have a greater impact on marital status, so it is important to weigh the groups by IPW, which can make the results generated by subsequent analyses more reliable. After IPW, each variable was equally comparable between the married and unmarried groups.

### Differences in the incidence of negative sleep consequences

There are discrepancies in the results of previous studies focused on sleep in military personnel. Seelig et al. found that during non-operational times, when service members work a standard duty day, military personnel tend to sleep less than the general civilian population (49). However, previously reported insomnia rates among United States military personnel range from 11.2 to 16.3%, which is similar to the prevalence among residents (50, 51). The results of a meta-analysis of four diverse United States Army units showed that military personnel had PQSI scores of approximately 5 points (52), indicating that participants in the present study had generally good sleep quality (53). The prevalence of daytime sleepiness among the participant in this study was also lower than that in previous studies (54), as was the incidence of dysfunctional beliefs (55). Overall, the participants had better sleep quality, fewer dysfunctional beliefs, and less daytime sleepiness compared to previous studies on the sleep quality of military personnel in the Navy and some difficult and remote areas. These differences may arise because our participants were younger, and the survey was conducted during a non-mission period when participants may have been in a more stable state of life and work (50).

### Marital status differences in sleep quality

Contrary to expectations, results revealed that married individuals had worse subjective sleep quality and shorter sleep duration, and the results of logistic regression also showed that married individuals were 1.4 times more likely to have poor sleep quality than unmarried individuals. Many previous research findings support the positive health effects of marriage, that is, its marital protection effect (36). Notably, one view of marriage suggests that the greater resources gained through long-term commitment and shared destiny can be attributed to the better health of married individuals (56). However, the impact of marriage on sleep is divisive, especially among the military population (14, 16, 57, 58). How does marital status influence sleep quality? A semi-structured focus group questionnaire study on Polish firefighters revealed that married firefighters are burdened with more family responsibilities and may be inclined to sacrifice sleep for family responsibilities. At the same time, the support of a spouse can encourage sleep. Conversely, single firefighters enjoy more adequate rest and conditioning time (59). Military personnel, similar to firefighters, bear a tremendous professional burden and face more than usual difficulties in balancing family and work, hence they are likely to face similar situations (60). In addition, our results suggest that married military personnel may have less sleep duration which may be due to the need to care for their families. Results also indicate that married individuals are more efficient sleepers, use fewer medications, and have less daytime dysfunction than unmarried individuals, suggesting that despite poor overall sleep quality scores, married military personnel can compensate for the demands of daytime work by improving their sleep efficiency. Another possible explanation for the results is that sleeping with a spouse who develops sleep apnea may interfere with the individual’s sleep (20). The lack of investigation of the physical condition of the spouse in this study warrants caution in the interpretation of the results. Future studies should consider more factors, including marital quality, changes in marital status, and spouse’s physical condition.

Compared to the unmarried group, married participants were more likely to have daytime sleepiness. Many previous studies have shown that being married is associated with an increased risk of developing daytime sleepiness (61–63). As mentioned above, this may be due to the need for married military personnel to devote more energy to family issues, resulting in less energy during the day (59).

To our surprise, we found that dysfunctional sleep beliefs were more severe in the married population, both in total scores and scores of each dimension. Compared with the relevant scores with those reported by Jin et al. in a college student population, we found that the mean DBAS-16 scores in this study were higher in unmarried participants but lower in married ones, indicating that the DBAS in the married group was more severe than in the Chinese general population (64). Previous research has suggested that family environment has an important influence on the formation of sleep beliefs (65). The current results indicate that sleep beliefs in military personnel are not protected by marriage, suggesting that there may be other factors that influence sleep beliefs (44). For example, in the military population, being married does not necessarily mean that you can live with your partner and receive face-to-face support, and arguments with your partner may exacerbate dysfunctional sleep beliefs (66). In addition, simply grouping as married or unmarried can also cause differences in results.

### The effect of living apart or not on sleep

Military personnel often have to live apart from their spouses, due to deployment or mission requirements, even if they are married. In contrast, unmarried service members often live collectively at their duty station. Therefore, a generalized analysis of married and unmarried groups separately may result in bias, and living apart or not deserves our further attention. When examining living apart or not and its relationship to sleep-related outcomes, a different pattern emerged. The sleep quality of married people living together did not differ much from that of unmarried people, whereas married people living separately had significantly poorer sleep quality. In other words, it is the difference between married but living apart and unmarried that causes the difference between married and unmarried to become larger. Living with a spouse, rather than married status, has a protective effect on the sleep of military personnel, suggesting that face-to-face family support may be more important and effective than remote support.

Results of the subgroup analysis also revealed differences in other sleep-related outcomes. Firstly, those living together exhibited higher odds of daytime sleepiness, whereas the odds of living apart were lower, indicating that married people who live together often need to sacrifice their sleep time to be with their families and share family responsibilities (66). These findings are consistent with other studies that suggest that obtaining a bed partner through marriage negatively affects daytime performance (67).

Moreover, the ORs for DBAS in the married and unmarried populations may have resulted from a disproportionately large OR in married living apart, with a smaller probability of having dysfunctional sleep beliefs in the comparison between married living together and unmarried populations. Married living together is a protective factor for the development of dysfunctional sleep beliefs, whereas married living apart is a greater risk factor and should be viewed separately when drawing conclusions. A reasonable explanation is that beliefs about sleep, similar to other beliefs, are formed over time in daily life and are influenced by those around them (68). Moreover, the family commitment received by co-resident married people may be one of the reasons why they exhibit better sleep beliefs (34). Being married but living apart may also cause affective reactions, such as missing their spouse and children, which can lead to dysfunctional beliefs (69). To improve the sleep quality and beliefs of military personnel, in addition to cognitive behavioral intervention (55), support from cohabitants may be critical. Considering the current status of the military population, care from comrades may be a potential target for intervention for both unmarried and married but living apart under conditions where spousal care is not available. Also, increasing the frequency of contact with family members for married but living apart may contribute to the development of health beliefs. Further research is needed to explore the factors that influence sleep beliefs among military personnel.

### Other demographic factors on sleep

Other demographic factors also affected military sleep. Age barely changed the OR value in the three models, suggesting that age was an independent protective factor for poor sleep outcomes. Consistent with the findings of a study that included both younger and older individuals during the pandemic quarantine, there was a trend toward lighter dysfunctional sleep beliefs among older individuals than younger individuals (70–72). The ability of stress to promote insomnia is well known, and Blake et al. proposed a “biopsychosocial” model that explains the parallel and interactive effects of sleep and dysfunctional beliefs and attitudes as psychological factors for insomnia, anxiety, and depression (73). Similar to our findings, family stress can promote the development of poor sleep quality, daytime sleepiness, and other co-occurring psychological problems. Participants with good family economic status were less likely to have poor sleep quality. However, a better family economy was also associated with more daytime sleepiness, which highlights that heavy financial burdens can disrupt military personnel’s sleep but at the same time force them to stay awake during the day. These findings are inconsistent with those of previous studies that have shown a positive association between family finances and sleep quality (74). Furthermore, the better the comradeship, the worse the quality of sleep, suggesting that male service members may have difficulty balancing work and life, that the burden of family and work can negatively affect their sleep, and that it is especially important to focus on those who have strong relationships with their comrades.

### Limitation and future directions

There are some shortcomings in our study. First, we only explored the effect of current marital status on sleep-related outcomes without conducting a long-term observational cohort study. The formation of sleep pattern is a chronic process, and it may be more meaningful to explore the relationship between other marital factors (e.g., marriage duration, marital transition, marital quality) and sleep. Second, the survey of demographic data is determined by several single-choice questions, which may lead to low validity of the measurement, but it can also reflect the impact of subjective indicators on the outcomes. Subsequent studies should include more factors related to marriage and use scales with a higher validity for measurement. Third, the sample for this study was limited to a younger group of male military personnel and no participants were divorced or widowed, which may create bias. Subsequent studies could select a more elderly group and explore how the results would be changed when it comes to “divorced” or “widowed.” In addition, more studies that include females are needed in the future to explore the effect of gender on this finding. Finally, the lack of the investigation about the mechanism underlying the effect of marital status on sleep remained to be solved in the future. Some studies have suggested that attachment orientation may have an impact on sleep quality through relationship-specific security and negative affect (75), but much work remains to be done (76).

## Conclusion

This cross-sectional study examined the effects of marital status on sleep quality, daytime sleepiness, and sleep beliefs. To our knowledge, this study is the first to explore the relationship between marital status and sleep-related outcomes among military personnel. The findings showed that married participants were more likely to have poor sleep quality, daytime sleepiness, and dysfunctional sleep beliefs than unmarried participants. Married living together is a risk factor for daytime sleepiness among male military personnel while a protective factor for subjective poor sleep quality and the development of dysfunctional sleep beliefs. Married living apart is a great risk factor for dysfunctional beliefs. The findings of this study fill a gap in the effect of marital status on sleep in the military population, which suggest that we can implement more effective interventions for sleep quality and dysfunctional sleep beliefs among married military personnel, especially for those married but living apart from their spouses. Increasing the frequency of contact with family members for married but living apart may contribute to the development of health beliefs. Further research on the effects of marital status on sleep will help troops to efficiently intervene in sleep problems and develop individualized intervention programs.

## Data availability statement

The raw data supporting the conclusions of this article will be made available by the authors, without undue reservation.

## Ethics statement

The studies involving human participants were reviewed and approved by Medical Research Ethics Committee of the Naval Medical University. The patients/participants provided their written informed consent to participate in this study.

## Author contributions

XG, YM, and HL contributed to the writing of this letter and the statistical analysis of this letter, who are co-first author. YT leaded the whole study, including putting forward this study, carrying out the study, and was the corresponding author. YL, YX, RZ, JX, HW, SX, WC, LX, and TS contributed to perform the investigation and collection of all data. All authors were accountable for all aspects of the work in ensuring that questions related to the accuracy or integrity of any part of the work are appropriately investigated and resolved.

## Funding

The authors disclosed receipt of the following financial support for the research, authorship, and/or publication of this article: Navy 13th Five-Year Dual Construction Project (2020SZ16); 2020 Military Logistics Scientific Research Program Projects (20BJZ09); and Changzheng Hospital Pyramid Talent Project (2020) of Naval Medical University.

## Conflict of interest

The authors declare that the research was conducted in the absence of any commercial or financial relationships that could be construed as a potential conflict of interest.

## Publisher’s note

All claims expressed in this article are solely those of the authors and do not necessarily represent those of their affiliated organizations, or those of the publisher, the editors and the reviewers. Any product that may be evaluated in this article, or claim that may be made by its manufacturer, is not guaranteed or endorsed by the publisher.
