# Supplementary material for: Marital status and living apart affect sleep quality in male military personnel: a study of the China’s Navy during COVID-19
Source: Front Psychiatry. 2023 Jul 27;14:1178235. doi: 10.3389/fpsyt.2023.1178235 (PMC10412872; doi:10.3389/fpsyt.2023.1178235)
Supplement: Supplementary file 1 [file Table_1.docx]

| \| Table S1: Prevalence and mean scores of sleep-related outcomes by marital status before IPW \| \| \| \| \| \| --- \| --- \| --- \| --- \| --- \| \|  \| **Total** \| **Unmarried** \| **Married** \| **P** \| \| **N** \| 1784 \| 1432 \| 353 \|  \| \| **Prevalence of poor sleep quality (%)** \| 261(14.6) \| 182(12.7) \| 79 (22.4) \| <0.001 \| \| **Total PSQI** \| 3.55(2.87) \| 3.29 (2.78) \| 4.59 (3.01) \| <0.001 \| \| Subjective sleep quality \| 0.68(0.64) \| 0.64 (0.62) \| 0.86 (0.68) \| <0.001 \| \| Sleep latency \| 0.74(0.77) \| 0.69 (0.76) \| 0.96 (0.78) \| <0.001 \| \| Sleep duration \| 0.54(0.59) \| 0.50 (0.57) \| 0.70 (0.62) \| <0.001 \| \| Sleep efficiency \| 0.31(0.62) \| 0.28 (0.62) \| 0.41 (0.73) \| <0.001 \| \| Sleep disturbance \| 0.62(0.57) \| 0.58 (0.56) \| 0.78 (0.58) \| <0.001 \| \| Use of sleep medication \| 0.02(0.23) \| 0.02 (0.22) \| 0.02 (0.25) \| 0.977 \| \| Daytime dysfunction \| 0.63(0.82) \| 0.58 (0.80) \| 0.85 (0.87) \| <0.001 \| \| **Prevalence of sleepiness (%)** \| 463(26.0) \| 351 (24.5) \| 112 (31.7) \| 0.007 \| \| **Prevalence of severe sleepiness (%)** \| 91(5.1) \| 83 (5.8) \| 8 (2.3) \| <0.001 \| \| **Total ESS** \| 7.54(4.80) \| 7.34 (4.96) \| 8.34 (3.96) \| <0.001 \| \| **Prevalence of DBAS (%)** \| 80(4.5) \| 75 (5.2) \| 5(1.4) \| <0.001 \| \| **Average score of DBAS-16** \| 3.37(0.72) \| 3.38(0.75) \| 3.32 (0.54) \| 0.160 \| \| **Total DBAS-16** \| 53.89(11.46) \| 54.08 (12.04) \| 53.13 (8.69) \| 0.160 \| \| Consequences of insomnia \| 16.55(4.30) \| 16.68 (4.47) \| 16.01 (3.51) \| 0.009 \| \| Worry/helplessness about sleep \| 21.33(4.93) \| 21.37 (5.16) \| 21.17 (3.87) \| 0.498 \| \| Expectations for sleep \| 4.07(1.79) \| 4.14 (1.83) \| 3.80 (1.57) \| 0.001 \| \| Medication \| 11.94(2.41) \| 11.89 (2.50) \| 12.14 (1.97) \| 0.086 \| |
| --- | --- | --- | --- | --- | --- | --- | --- | --- | --- | --- | --- | --- | --- | --- | --- | --- | --- | --- | --- | --- | --- | --- | --- | --- | --- | --- | --- | --- | --- | --- | --- | --- | --- | --- | --- | --- | --- | --- | --- | --- | --- | --- | --- | --- | --- | --- | --- | --- | --- | --- | --- | --- | --- | --- | --- | --- | --- | --- | --- | --- | --- | --- | --- | --- | --- | --- | --- | --- | --- | --- | --- | --- | --- | --- | --- | --- | --- | --- | --- | --- | --- | --- | --- | --- | --- | --- | --- | --- | --- | --- | --- | --- | --- | --- | --- | --- | --- | --- | --- | --- | --- | --- | --- | --- | --- | --- | --- | --- | --- | --- |
